# Autologous bone marrow-derived mononuclear cell therapy in three patients with severe asthma

**DOI:** 10.1186/s13287-020-01675-x

**Published:** 2020-05-01

**Authors:** Fabio S. Aguiar, André S. Melo, Ana Maria S. Araújo, Alexandre P. Cardoso, Sergio Augusto L. de Souza, Miquéias Lopes-Pacheco, Fernanda F. Cruz, Debora G. Xisto, Karina D. Asensi, Lanuza Faccioli, Anna Beatriz S. Salgado, Maria Carolina P. P. Landesmann, Regina C. S. Goldenberg, Bianca Gutfilen, Marcelo M. Morales, Patricia R. M. Rocco, Jose R. Lapa e Silva

**Affiliations:** 1 Institute of Thoracic Medicine, Clementino Fraga Filho University Hospital, Federal University of Rio de Janeiro, Rio de Janeiro, Brazil; 2 Department of Radiology, Federal University of Rio de Janeiro, Rio de Janeiro, Brazil; 3 Laboratory of Pulmonary Investigation, Carlos Chagas Filho Biophysics Institute, Federal University of Rio de Janeiro, Rio de Janeiro, Brazil; 4 National Institute of Science and Technology for Regenerative Medicine, Rio de Janeiro, Brazil; 5 Laboratory of Cellular and Molecular Physiology, Carlos Chagas Filho Biophysics Institute, Federal University of Rio de Janeiro, Rio de Janeiro, Brazil; 6 Laboratory of Cellular and Molecular Cardiology, Carlos Chagas Filho Biophysics Institute, Federal University of Rio de Janeiro, Rio de Janeiro, Brazil; 7 Department of Clinical Hematology, Clementino Fraga Filho University Hospital, Federal University of Rio de Janeiro, Rio de Janeiro, Brazil

**Keywords:** Asthma, Bone marrow mononuclear cells, Cell therapy, Autologous transplantation, Lung

## Abstract

**Background:**

Despite recent advances in understanding its pathophysiology and development of novel therapies, asthma remains a serious public health issue worldwide. Combination therapy with inhaled corticosteroids and long-acting β_2_-adrenoceptor agonists results in disease control for many patients, but those who exhibit severe asthma are often unresponsive to conventional treatment, experiencing worse quality of life, frequent exacerbations, and increasing healthcare costs. Bone marrow-derived mononuclear cell (BMMC) transplantation has been shown to reduce airway inflammation and remodeling and improve lung function in experimental models of allergic asthma.

**Methods:**

This is a case series of three patients who presented severe asthma, unresponsive to conventional therapy and omalizumab. They received a single intravenous dose of autologous BMMCs (2 × 10^7^) and were periodically evaluated for 1 year after the procedure. Endpoint assessments included physical examination, quality of life questionnaires, imaging (computed tomography, single-photon emission computed tomography, and ventilation/perfusion scan), lung function tests, and a 6-min walk test.

**Results:**

All patients completed the follow-up protocol. No serious adverse events attributable to BMMC transplantation were observed during or after the procedure. Lung function remained stable throughout. A slight increase in ventilation of the right lung was observed on day 120 after BMMC transplantation in one patient. All three patients reported improvement in quality of life in the early post-procedure course.

**Conclusions:**

This paper described for the first time the effects of BMMC therapy in patients with severe asthma, providing a basis for subsequent trials to assess the efficacy of this therapy.

## Background

Asthma is a chronic inflammatory disease that represents an increasing public health issue worldwide, affecting 1–18% of the population in different countries [1]. It is characterized by airflow obstruction with airway inflammation and hyper-responsiveness [2]. Most patients with asthma achieve disease control with a combination of inhaled corticosteroids and long-acting β_2_-adrenoceptor agonists (LABAs). However, a substantial proportion of patients with severe asthma are unresponsive to these treatments, experiencing unsuccessful control of their symptoms, pulmonary exacerbations, and accelerated deterioration of lung function [1, 2].

A growing body of evidence suggests that cell-based therapies hold therapeutic promise for patients with lung diseases. Bone marrow-derived mononuclear cells (BMMCs) and mesenchymal stromal cells (MSCs), administered either locally or systemically, have yielded favorable therapeutic outcomes in a wide spectrum of experimental models [3–5]. In animal models of allergic asthma, cell-based therapies reduced airway inflammation and remodeling significantly, thus improving lung function [4, 6–11]. Furthermore, we previously found that in a model of ovalbumin-induced allergic asthma, BMMCs induced better therapeutic responses compared to MSCs in some endpoint assessments, including reduction of alveolar collapse area and collagen fiber content in lung tissue [10]. Autologous BMMCs can also be transplanted on the same day of harvesting, thus avoiding additional costs related to cell culture procedures and potential host-recipient mismatch complications, such as graft-versus-host disease.

In early-stage clinical studies, BMMC therapy demonstrated a safety profile in patients with idiopathic dilated cardiomyopathy, ischemic stroke, or silicosis [12–14]. We described three patients with severe asthma unresponsive to conventional treatment (corticosteroids, LABA, muscarinic antagonists) and omalizumab who received autologous transplantation of BMMCs intravenously. These patients were followed up periodically for 1 year after the procedure. Endpoint assessments included physical examination, quality of life (QoL) questionnaires, imaging (computed tomography (CT), single-photon emission computed tomography (SPECT), and ventilation/perfusion scan), lung function tests, and a 6-min walk test (6MWT). BMMCs were characterized on the basis of a fibroblast colony-forming assay and expression of specific surface antigens by flow cytometry analysis.

## Material and methods

### Ethics statement

Patients were recruited from the Severe Asthma Reference Center at the Institute of Thoracic Diseases, Clementino Fraga Filho University Hospital (HUCFF), Federal University of Rio de Janeiro (UFRJ), Brazil. All patients were investigated and treated in accordance with the Global Initiative for Asthma (GINA) guidelines [1]. The patients also received standard care according to the institutional protocol of the HUCFF/UFRJ Pulmonary Division, before and after autologous transplantation of BMMCs. The study protocol and informed consent form were approved by the Brazilian National Research Ethics Committee (CONEP; study ID number: 06503212.3.0000.5257).

### Study population

Ten patients with severe asthma were initially screened for eligibility. The inclusion criteria were (1) age between 18 and 65 years; (2) uncontrolled asthma despite maximum therapy in the previous 6 months, as recommended by the GINA guidelines [1]; (3) unresponsiveness to immunobiological therapy with omalizumab; (4) a forced expiratory volume in 1 s (FEV_1_) < 80% on spirometry and a bronchodilator response at any time; and (5) informed consent to participate in the study. The exclusion criteria were (1) an infectious episode in the 4 weeks preceding enrollment; (2) other lung diseases, including active tuberculosis; (3) current or recent smoking (within 12 months of inclusion), characterized by tobacco intake greater than 10 pack-years; (4) any malignant neoplasm; (5) autoimmune diseases; (6) hematologic or cardiovascular diseases; (7) seropositivity for the human immunodeficiency virus; (8) pregnancy; and (9) participation in any other clinical trial. Based on these criteria, three women were included in the study.

### Bone marrow aspiration, cell separation, and labeling

The bone marrow was aspirated under local anesthesia from the posterior iliac crest as previously described [12, 13]. BMMCs were isolated by density gradient on Ficoll-Paque at 400×*g* for 30 min (Ficoll-Paque Plus 1.077, 1:2, Amersham Biosciences, São Paulo, Brazil), washed twice in saline, and resuspended in saline solution with 10% autologous serum. After washing and counting, a total of 2 × 10^7^ cells were labeled with technetium-99m (^99m^Tc) for tracking after infusion, as described elsewhere [12, 13]. Cell viability was assessed by the trypan blue exclusion test before and after labeling and was estimated to be greater than 93% in all cases. All procedures for cell preparation and labeling were carried out under sterile conditions in a laminar flow hood. Bacteriological analyses and cultures were also performed to exclude any contamination of the specimens.

### Flow cytometry analysis

Total bone marrow and BMMCs were characterized by flow cytometry using specific surface antigens. Briefly, cells were incubated for 20 min at room temperature with primary antibodies conjugated with fluorescein isothiocyanate (FITC), phycoerythrin (PE), allophycocyanin (APC), peridinin chlorophyll protein (PercP), phycoerythrin cyano 5 (PE-Cy5), and phycoerythrin cyano 7 (PE-Cy7). After staining, erythrocytes were lysed with BD (Becton Dickinson) FACS Lysing Solution. Data acquisition was performed on a FACS ARIA II (BD Biosciences) flow cytometer and analyzed in Infinicity software (Cytognos, Spain). The panel of markers tested included CD45 FITC (clone HI30, BD), CD13 PE (clone WM15, BD Pharmingen), CD11b APC (clone MEM-174, Exbio), CD34 FITC (clone 8G12, BD Biosciences), CD117 PE (clone YB5.B8, BD Pharmingen), HLA-DR PE-Cy5 (clone TU36, BD Pharmingen), CD45 APC (clone MEM-28, Exbio), CD64 FITC (clone 10.1, BD Pharmingen), CD34 PE (clone 8G12, BD Biosciences), CD14 PE (clone MφP9, BD Pharmingen), CD20 FITC (clone LT20, Exbio), CD10 PE (clone MEM-78, Exbio), CD19APC (clone HIB19, BD Pharmingen), CD45 APC-Cy7 (clone 2D1, BD Pharmingen), lineage cocktail 2 (LIN2, composed of CD3, CD14, CD19, CD20, and CD56; clones SK7, MφP9, SJ25C1, L27, and NCAM16.2, respectively, BD Biosciences), CD105 PE (clone 266, BD Pharmingen), CD90 PE-Cy5 (clone 5E10, BD Pharmingen), CD73 APC (clone AD2, BD Pharmingen), Lymphogram™ (composed of CD8 + CD19 FITC, CD3 + CD56 PE, and CD4 PE-Cy5; clones UCH-T4, HD37, 33-2-A3, C5.9, 13B8.2 respectively, Cytognos), CD36 PE (clone CB38, BD Pharmingen), and CD71 APC (clone M-A712, BD Pharmingen).

### Fibroblast colony-forming unit assay

A fibroblast colony-forming assay was performed to determine the presence of putative progenitor cells of mesenchymal lineages. After Ficoll-Paque centrifugation, mononuclear cells were counted and plated in triplicate in a 6-well plate. A total of 2 × 10^6^ cells was cultured in each well using high-glucose Dulbecco’s modified Eagle’s medium (DMEM) supplemented with 10% fetal bovine serum (Gibco) and 10^−6^ M hydrocortisone. After plating, cells were maintained in a humidified incubator (37 °C, 5% CO_2_) for 1 week without any manipulation. Then, 50% of the medium was replaced and cells were maintained under the same conditions for an additional week. At the end of this protocol, cells were stained with Giemsa and colonies were counted.

### BMMC transplantation and imaging

A 20-mL aliquot of the autologous BMMC solution (2 × 10^7^ cells labeled with ^99m^Tc) was injected into a peripheral vein of the upper arm of each patient. After the procedure, patients were monitored for 1 h and then transferred to the Nuclear Medicine Department for further analyses. Whole-body, planar, and tomographic scintigraphy was carried out 2 h after BMMC transplantation. For regional analysis in both the anterior (A) and posterior (P) images, rectangular regions of interest, equal in size, were drawn over the whole lung. Both lungs had six regions of interest: right upper, right middle, right lower, left upper, left middle, and left lower lung fields. Regional blood flow was evaluated by ^99m^Tc macroaggregated albumin (^99m^Tc-MAA) perfusion scintigraphy in each area of interest and calculated as previously described [15]. Patients were followed for a further 12 months after BMMC transplantation.

### Measured variables

Patient demographics, medical history, vital signs, routine laboratory tests (blood counts, coagulation tests, biochemical measurements, liver function tests), electrocardiogram, the Modified Borg Dyspnea Scale, and the 6-min walk test (6MWT) were assessed using standardized clinical report forms. Lung function tests, including spirometry, lung CT scans, and ventilation/perfusion scintigraphy, were also performed. Quality of life (QoL) was assessed with the Saint George’s Respiratory Questionnaire (SGRQ) [16], which is a standardized set of self-report measures for assessment of impaired health and perceived well-being in patients with lung diseases. Measurements were performed before BMMC transplantation (baseline) and at 7, 30, 60, 120, 180, and 360 days after the procedure.

## Results

### Bone marrow aspiration and cellular characterization

**Fig. 1:**
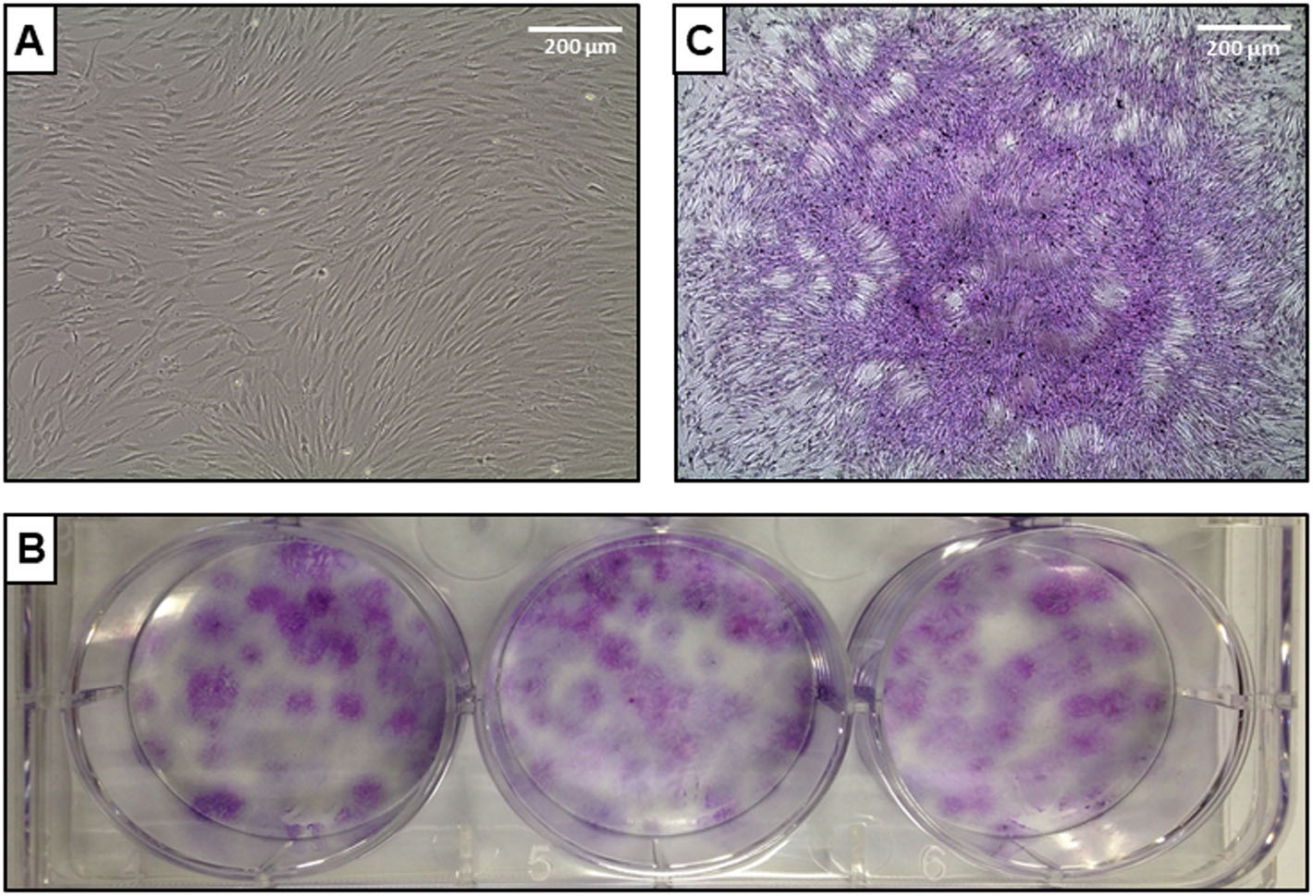
Representative images of fibroblast colony-forming unit assay. **a** Primary culture of BMMCs 14 days after plating. **b** Macroscopic image of cell culture plate after Giemsa staining. **c** Microscopic image of a single stained colony after 14 days in culture

The bone marrow was aspirated from the posterior iliac crest of patients (volume = 160.5 ± 51.7 mL; number of collected cells = 8.8 × 10^7^ ± 1.0 × 10^7^; cell viability = 94.3 ± 1.2%) and prepared for separation, characterization, and transplantation. Tables 1 and 2 depict, respectively, cell populations in total bone marrow and BMMCs obtained after separation. The total bone marrow was mainly composed of neutrophils (~ 70%), while a significant increase in mononuclear cell populations, including T lymphocytes (35.5%), helper T cells (13%), and cytotoxic T cells (9.5%), was observed after density gradient on Ficoll-Paque. The presence of putative progenitor cells of mesenchymal lineages was confirmed by a fibroblast colony-forming assay, with approximately 20 colonies counted per 10^6^ BMMC seeded (Fig. 1).

**Table 1:** Flow cytometry analysis of total bone marrow cells

CD markers	Phenotype	Percentage
CD45^low^CD34^high^SSC↓	Hematopoietic stem cells	0.88
CD45^low^CD34^+^CD19^+^	B cell progenitors	3.26
CD45^+^CD34^−^CD64^+^CD14^−^	Promonocytes	0.88
CD105^+^CD90^high^CD73^+^CD45^−^CD34^−^	Mesenchymal stromal cells	0.1
FSC↓SSC↓CD45^−^CD71^+^CD36^+^	Erythroblasts	8.1
CD45^+^CD34^−^CD64^+^CD14^+^	Monocytes	2.92
CD45^+^CD34^−^CD3^+^	T lymphocytes	14.13
CD45^+^CD3^+^CD4^+^	Helper T cells	5.16
CD45^+^CD3^+^CD8^+^	Cytotoxic T cells	4.33
CD45^+^CD3^−^CD19^+^	B cells	3.2
CD45^+^CD56^+^	NK cells	1.2
CD45^low^SSC↑	Neutrophils	69.56

*Abbreviations*: *CD* cluster of differentiation, *FSC* forward scatter, *NK* natural killer, *SSC* side scatter

**Table 2:** Flow cytometry analysis of BMMC cell fraction

CD markers	Phenotype	Percentage
CD45^low^CD34^high^SSC↓	Hematopoietic stem cells	1.83
CD45^low^CD34^+^CD19^+^	B cell progenitors	5.6
CD45^+^CD34^−^CD64^+^CD14^−^	Promonocytes	4.27
CD105^+^CD90^high^CD73^+^CD45^−^CD34^−^	Mesenchymal stromal cells	0.07
FSC↓SSC↓CD45^−^CD71^+^CD36^+^	Erythroblasts	6.46
CD45^+^CD34^−^CD64^+^CD14^+^	Monocytes	9.6
CD45^+^CD34^−^CD3^+^	T lymphocytes	35.53
CD45^+^CD3^+^CD4^+^	Helper T cells	12.96
CD45^+^CD3^+^CD8^+^	Cytotoxic T cells	9.46
CD45^+^CD3^−^CD19^+^	B cells	5.6
CD45^+^CD56^+^	NK cells	4.46

*Abbreviations*: *CD* cluster of differentiation, *FSC* forward scatter, *NK* natural killer, *SSC* side scatter

### Clinical presentation

Three individuals with severe asthma were included in the study and periodically followed up for 1 year after BMMC transplantation (2 × 10^7^ cells/patient). All were women and had a mean age of 55 years (range 50–58 years) and body mass index (BMI) of 29.5 kg m^-2^ (range 22.4–34.1 kg.m^-2^) at intervention. Although all three patients had severe asthma, considerable heterogeneity in baseline clinical parameters and signs was observed among them (Table 3).

**Table 3:** Clinical parameters

		Visit (days)						
Patient	Parameters	D–28 (baseline)	D + 7	D + 30	D + 60	D + 120	D + 180	D + 360
1	FVC (L)	2.23	2.18	2.04	2.06	1.87	2.05	1.89
	FVC (%)	76	75	70	70	64	70	65
	FEV_1_, pre-BD (L)	1.5	1.6	1.48	1.57	1.5	1.56	1.46
	FEV_1_ (%)	62	66	61	65	62	65	61
	6MWT (m)	273	273	291	276	240	269	294
2	FVC (L)	1.99	2.00	2.40	1.72	1.78	1.85	1.77
	FVC (%)	74	74	89	64	66	68	66
	FEV_1_, pre-BD (L)	0.88	0.95	1.08	0.65	0.71	0.76	0.66
	FEV_1_ (%)	41	44	50	30	33	35	31
	6MWT(m)	412	*	380	255	310	176	324
3	FVC (L)	1.89	2.10	1.87	1.99	1.94	1.68	1.86
	FVC (%)	64	72	64	68	66	57	64
	FEV_1_, pre-BD (L)	0.83	0.85	0.77	0.87	0.86	0.78	0.85
	FEV_1_ (%)	35	36	33	37	37	33	37
	6MWT (m)	354	390	380	450	441	360	390

*Abbreviations*: *6MWT* 6-min walk test, *BD* bronchodilator, *FEV*_*1*_ forced expiratory volume in 1 s, *FVC* forced vital capacity

*Missing data

**Fig. 2:**
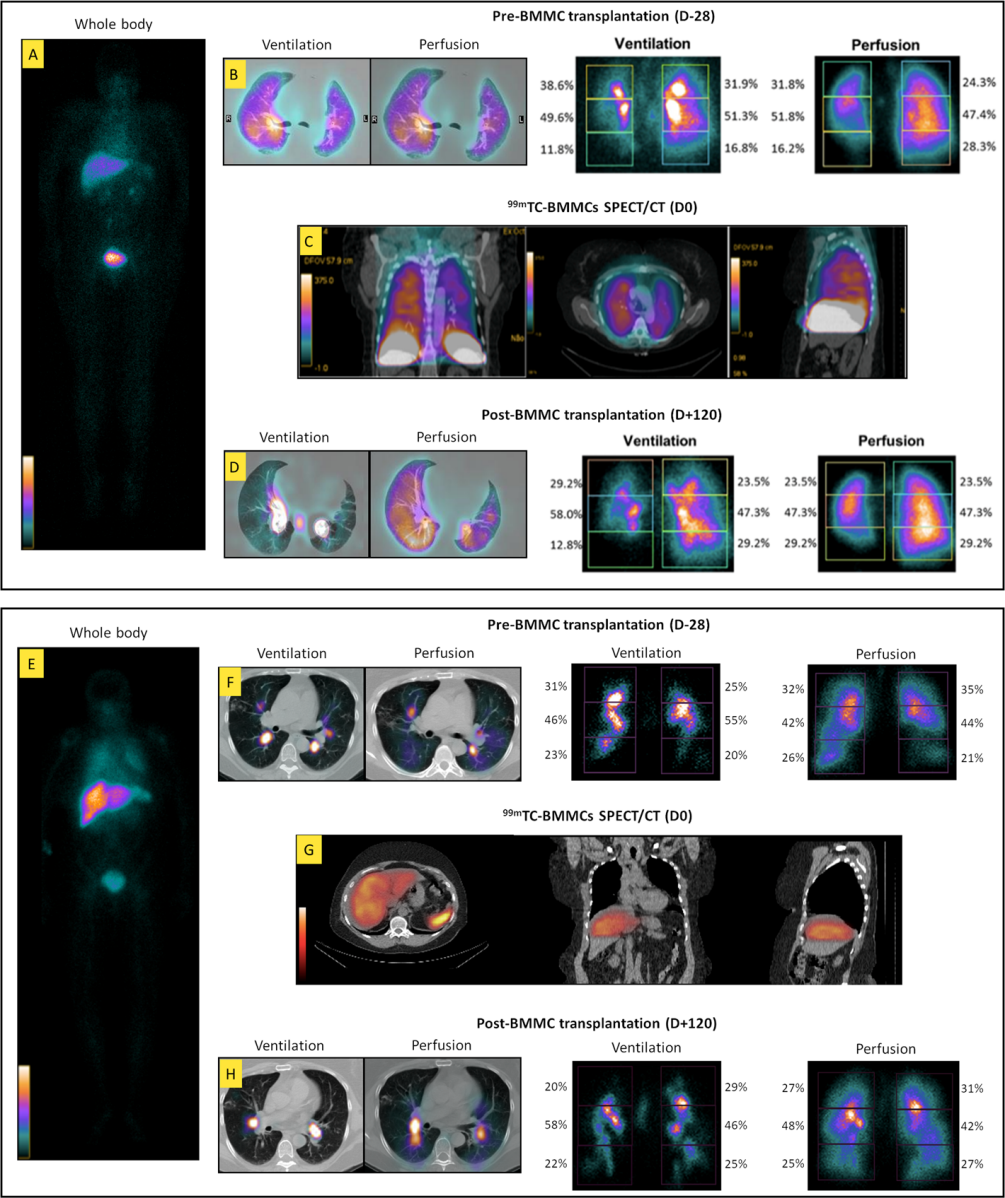
Imaging of patients 1 (upper panel) and 2 (lower panel). **a**, **e** Whole-body (WB) scintigraphy 2 h after intravenous administration of ^99m^Tc-BMMCs. Uptake found in the liver, lungs, heart, blood pool, kidney, and bladder. **b**, **f** Lung ventilation/perfusion SPECT/CT 28 days before BMMC transplantation. **c**, **g**^99m^Tc-BMMC SPECT/CT demonstrating liver and lung uptake 2 h after BMMC transplantation. **d**, **h** Lung ventilation/perfusion SPECT/CT 120 days after BMMC transplantation

**Fig. 3:**
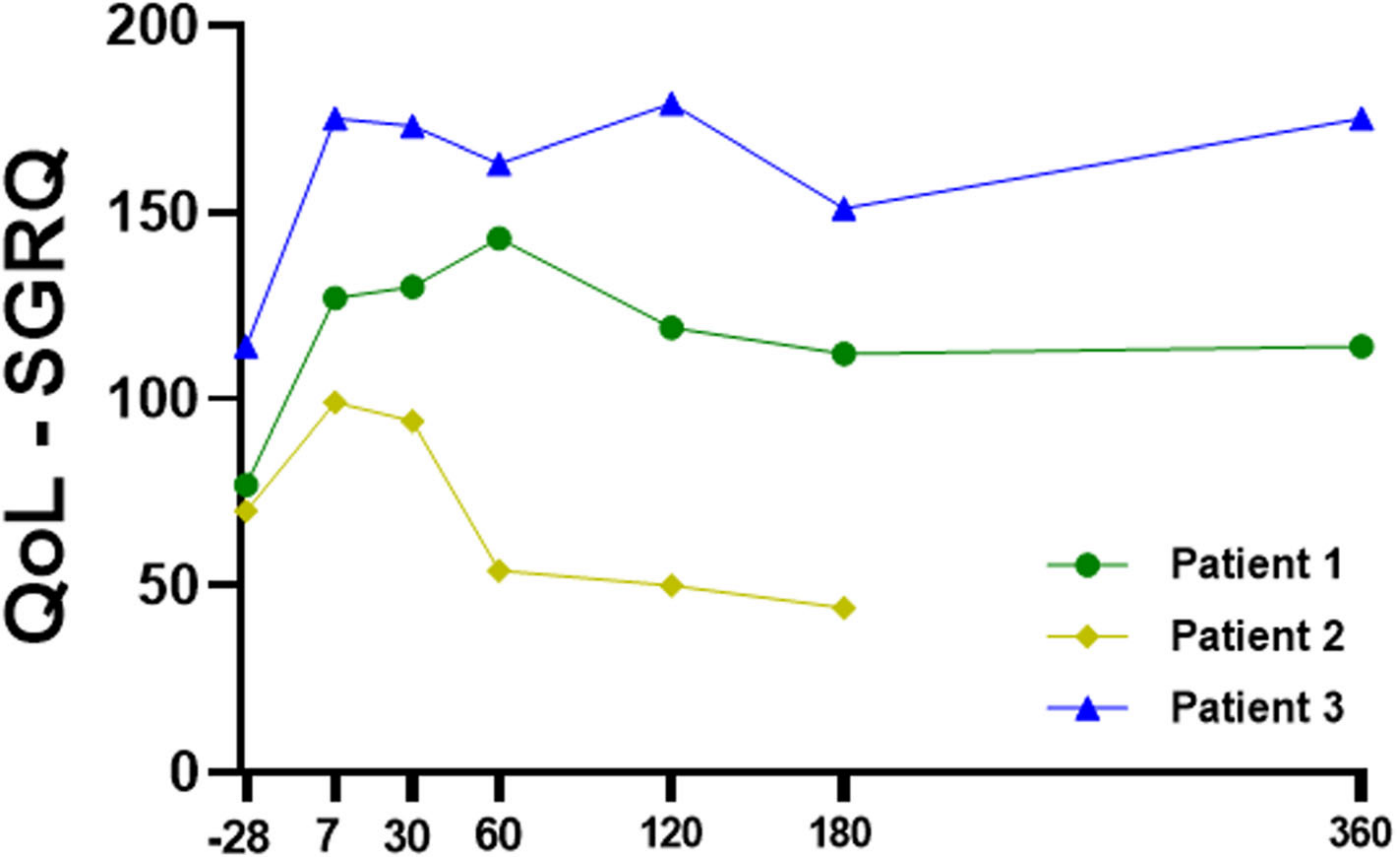
Evaluation of self-perceived quality of life (QoL) over the 1-year follow-up period, as measured by St. George’s Respiratory Questionnaire (SGQR)

*Patient 1* was a 50-year-old woman (height 1.55 m, weight 82 kg, BMI 34.1 kg m^-2^) with severe steroid-dependent allergic asthma who demonstrated no therapeutic response to omalizumab. The patient had never smoked. Disease onset had occurred in early childhood, with remission from age 15–25. The patient had a history of severe pulmonary exacerbations requiring mechanical ventilation and tracheostomy, despite regular treatment with high-dose oral and inhaled corticosteroids and LABA. In August 2015, the patient underwent bone marrow aspiration and received a single dose of autologous BMMCs intravenously. Measured variables were performed before treatment and periodically up to 1 year after BMMC transplantation (Table 3). Whole-body ^99m^Tc-BMMC scintigraphy was performed 2 h after administration and demonstrated a normal biodistribution of labeled cells, with greater uptake in the liver, lungs (especially the right), heart, blood pool, kidneys, and bladder (Fig. 2). Over the following days, the patient experienced improvement of respiratory symptoms but developed sinus tachycardia, requiring a reduction in LABA dose. A mild increase in ventilation of all zones of the right lung was also observed 120 days after BMMC transplantation. Asthma control was successfully achieved with half of the prior dose of inhaled corticosteroids and LABA for up to 12 months after BMMC transplantation. Self-perceived QoL score significantly improved on day 7 after the procedure and remained steady over 1 year of follow-up (Fig. 3). Lung function and 6MWD measurements remained stable throughout.

*Patient 2* was a 58-year-old woman (height 1.53 m, weight 75 kg, BMI 32.0 kg m^-2^) with severe steroid-dependent allergic asthma who also failed to respond to omalizumab. Disease onset had occurred in early childhood; there had been no remission. The patient had mild bronchiectasis without chronic infection and had never smoked. IgE testing for *Aspergillus fumigatus* was negative on multiple occasions. The patient was on regular high-dose oral and inhaled corticosteroids plus LABA and had a history of multiple hospital admissions due to pulmonary exacerbations. In November 2015, bone marrow aspiration was performed, and the patient received a single dose of autologous BMMCs intravenously and was periodically followed for a year (Table 3). Corticosteroids and LABA were continued. Whole-body ^99m^Tc-BMMC scintigraphy performed 2 h after cell infusion demonstrated a normal biodistribution of labeled cells, with greater uptake in the liver, lungs, heart, blood pool, kidneys, and bladder (Fig. 2). Thirty days after the procedure, asthma control was achieved, and the patient demonstrated better exercise tolerance as well as a reduction in corticosteroid requirements. During the following months, the patient had three pulmonary exacerbations, each requiring an increase in the dose of oral corticosteroids and prescription of antibiotics. No significant changes in ventilation or perfusion from baseline were observed on day 120 post-BMMC transplantation. Over the 1-year follow-up period, her asthma remained partially controlled. Lung function, 6MWD, and self-perceived QoL score improved slightly up to 30 days after BMMC transplantation (Table 3, Fig. 3). On day 60, these parameters declined, which remained stable thereafter.

*Patient 3* was a 58-year-old woman (height 1.58 m, weight 56 kg, BMI 22.4 kg m^-2^) with severe non-allergic asthma. Disease onset occurred at age 47; the patient had never smoked. She had dyspnea with minimal exertion despite regular treatment with high-dose inhaled corticosteroids, LABA, and a long-acting muscarinic antagonist. Severe obstruction was also observed on spirometry, with a positive bronchodilator test. In December 2016, the patient underwent bone marrow aspiration and received a single dose of autologous BMMCs intravenously. Lung function remained stable throughout the follow-up period. Asthma control, QoL score, and 6MWD improved slightly over the first 30 days after BMMC transplantation and remained stable up to day 180 (Table 3, Fig. 3). Thereafter, a mild increase in breathlessness was reported, but without the need to change baseline treatments. At 1-year follow-up, asthma symptoms were partially controlled, and both self-perceived QoL and 6MWD had improved from baseline.

In the first trimester of 2020, all patients were alive, with a significant reduction in the number of hospital admissions during their respective follow-up periods compared to before BMMC transplantation. Patient 2 had a pneumonia episode in 2019, however, recovered well. More recently, both patients 1 and 2 were using only an oral inhalation solution of formoterol fumarate plus budesonide, while patient 3 was using two oral inhalation solutions (formoterol fumarate/beclometasone and tiotropium bromide).

## Discussion

This case series describes the effects of a single intravenous dose of autologous BMMCs in three patients with severe asthma. The procedure was well tolerated, and no adverse events related to BMMC transplantation were observed during 1-year follow-up. Although these patients had heterogenous clinical features and signs at baseline, no significant changes were found in lung function tests after BMMC transplantation. Nevertheless, patients 1 and 3 experienced an improvement in self-perceived QoL score in the early post-procedure course, which remained stable throughout the follow-up period. Patient 2 also exhibited an improvement in self-perceived QoL in the early course after BMMC transplantation; however, this patient had a pulmonary exacerbation episode, which resulted in the worsening of QoL scores on day 60, remaining stable thereafter up to day 180. Total bone marrow cells and BMMCs of all patients were characterized using a defined panel of phenotypic markers. Furthermore, the functional characterization of fibroblastic-like cells was confirmed by the presence of putative progenitor cells of mesenchymal lineages.

Preclinical data have demonstrated the great potential of cell-based therapies in modulating inflammatory parameters, including recruitment and polarization of immune cells and production of cytokines/chemokines and growth factors [3–5]. Although MSC administration has been more extensively investigated for a wide range of diseases, we specifically evaluated the effects of BMMC transplantation in patients with severe asthma, as these cells were associated with greater reduction in lung tissue abnormalities and fibrosis in a murine model of ovalbumin-induced allergic asthma [10]. Furthermore, in a model *Aspergillus* hyphal extract-induced allergic asthma, reduction in inflammation and airway hyper-responsiveness was triggered by CD11b^+^ (monocytes, macrophages, dendritic cells) and Sca-1^+^ (MSCs) cells present in the pool of BMMCs. Such findings demonstrated that therapeutic effects were resulting from the balance between cell types rather than strictly from MSCs [6]. Autologous BMMCs can also be administered on the same day of harvesting, thus preventing further costs related to cell culture procedures and potential complications of human leukocyte antigen mismatch. A peripheral vein was used for BMMC transplantation, since the procedure could be easily performed without the need of intubation for bronchoscopy. Intravenous administration has demonstrated a good safety profile for delivery of either BMMCs or MSCs in experimental models [6, 7, 9, 17–20]. Even though a systemic route was used in the cases described herein, these cells are efficiently delivered to the lungs as they are subject to a first-pass effect [21].

The best cell dosing strategy to achieve optimal therapeutic outcomes in respiratory diseases is still unknown, and only a few dose-escalation clinical trials have been performed to date [3–5]. We used a fixed dose of 2 × 10^7^ BMMCs per patient, regardless of body weight; this dose was selected on the basis of clinical studies previously evaluating the safety and feasibility of BMMCs in patients with silicosis or ischemic stroke [12, 13]. Moreover, we aspirated the volume of the bone marrow (~ 150 mL) from which the maximum number of cells was harvested and injected. During the follow-up period, one patient demonstrated early improvement in self-perception of QoL, followed by a pulmonary exacerbation episode some months later, which resulted in a decline in QoL score. These adverse events were most likely related to disease progression and severity, as this patient already had a history of multiple hospital admissions due to pulmonary exacerbations. The remaining two patients demonstrated improvement in QoL score which remained throughout the study, although no therapeutic effects on lung function were noted. The safety profile found in this early-stage study is encouraging for future evaluations of whether administration of increasing doses of BMMCs may result in improved lung function without eliciting adverse effects in patients with severe asthma. Furthermore, a single dose of either BMMCs or MSCs has demonstrated therapeutic effects in experimental studies, but repeated cell-based therapy yielded greater effects—either by preventing disease progression or by further mitigating inflammation and remodeling—in animal models of silicosis, elastase-induced emphysema, and house dust mite-induced allergic asthma [7, 22, 23]. Future large-scale trials should be conducted to comparatively determine the potential efficacy of single-dose versus repeated cell-based therapy in patients with severe asthma.

To track BMMCs after systemic administration, cells were labeled with ^99m^Tc. This radioisotope has a short half-life (approximately 6 h) but allows efficient cell labeling for early-course tracking after administration, as well as a lower radiation burden for BMMCs and patients compared to other isotopes [13, 24, 25]. Although the chemical compounds used in labeling may cause cell damage, regardless of which technique is used, we did not observe any significant reduction in either BMMC or MSC viability in previous experimental and clinical studies using ^99m^Tc [12, 17, 18, 20]. Two hours after systemic administration of ^99m^Tc-BMMCs, these cells were localized in several organs, but a considerable uptake was observed in the lungs, possibly due to circulating chemoattractant mediators and first-pass effects. It remains to be elucidated whether and for how long BMMCs would persist in the lungs and other organs, as therapeutic effects of cell-based therapy in experimental models have been attributable to paracrine/endocrine actions without the need of cell engraftment [3–5].

No deaths or adverse events related to BMMC transplantation occurred during the procedure, nor during the follow-up period. Patients 1 and 3 reported improvement in QoL scores in the early post-procedure course, and their clinical features remained stable over 1-year follow-up. Although lung function and 6MWT did not improve significantly, a mild increase in ventilation of the right lung was observed in patient 1 120 days after BMMC transplantation. Interestingly, this patient also exhibited better baseline lung function values, which suggests that therapeutic outcomes might differ depending on disease severity during and after the intervention.

Our group is the first evaluating the effects of a single dose of BMMCs in patients with severe asthma. Limitations of this study include the small sample size, lack of a placebo-controlled, randomized design, and measurements of inflammatory biomarkers. Furthermore, as our primary aim was to evaluate the safety of a single dose of BMMCs, the design was underpowered for efficacy. Although a 1-year follow-up period is not long enough to enable definitive conclusions, the safety profile of BMMC transplantation observed herein is encouraging to pursue large-scale clinical trials in patients with severe asthma.

## Conclusion

A single intravenous dose of autologous BMMCs appears to be safe and improves self-perceived QoL in the early course after the procedure. These results provide a basis for subsequent clinical investigations of BMMCs or even other cell types in patients with severe asthma.

## Data Availability

The data that support the findings of this study are available from the corresponding author upon reasonable request.
